# Inferring chromatin accessibility during murine hematopoiesis through phylogenetic analysis

**DOI:** 10.1186/s13104-023-06507-8

**Published:** 2023-09-19

**Authors:** Kanako O. Koyanagi

**Affiliations:** Faculty of Information Science and Technology, Hokkaido University, Sapporo, Hokkaido Japan

**Keywords:** Cell differentiation, Chromatin accessibility, Phylogenetic analysis, Ancestral state estimation, Murine hematopoiesis

## Abstract

**Objective:**

Diversification of cell types and changes in epigenetic states during cell differentiation processes are important for understanding development. Recently, phylogenetic analysis using DNA methylation and histone modification information has been shown useful for inferring these processes. The purpose of this study was to examine whether chromatin accessibility data can help infer these processes in murine hematopoiesis.

**Results:**

Chromatin accessibility data could partially infer the hematopoietic differentiation hierarchy. Furthermore, based on the ancestral state estimation of internal nodes, the open/closed chromatin states of differentiating progenitor cells could be predicted with a specificity of 0.86–0.99 and sensitivity of 0.29–0.72. These results suggest that the phylogenetic analysis of chromatin accessibility could offer important information on cell differentiation, particularly for organisms from which progenitor cells are difficult to obtain.

**Supplementary Information:**

The online version contains supplementary material available at 10.1186/s13104-023-06507-8.

## Introduction

Cell differentiation is important for understanding how multicellular organisms develop based on their genetic programs. Recent high-throughput sequencing technologies and single-cell omics have revolutionized the way it is studied. To illustrate, recent advances in single-cell RNA-seq analysis have allowed researchers to infer cellular differentiation trajectories, which could be interpreted as a proxy for cellular progression along cellular differentiation pathways [1, 2]. However, this analysis requires cells in different states during the differentiation process, including stem cells and progenitor cells, to order them along pseudo-time. Considering that tissue stem cells and progenitor cells are typically rare and difficult to identify experimentally [3, 4], important processes involved in intermediate progenitor states might not be known from the analysis. To address this problem, previous studies based on bulk transcriptomes have applied phylogenetic analysis; phylogenetic analysis can infer not only tree topology—corresponding to the cell differentiation hierarchy [5]—but also ancestral states—corresponding to the states of the differentiating intermediate progenitor cells. Based on phylogenetic analysis of terminally differentiated mature cells, Kin et al. inferred the expression pattern of differentiating intermediate progenitor cells [6].

Epigenomes reflect the expression status of genes and contain information not only on gene regions but also on *cis*-regulatory regions [7]. Thus, the transition in epigenetic states during cell differentiation can provide further insight into the underlying mechanisms of cell differentiation. Epigenomes are somatically heritable and change during cellular differentiation, showing diversity among cell types [7]. Previous studies have shown that phylogenetic analysis of epigenomic information such as DNA methylation [8–10] and histone modification [11–13] of terminally differentiated cells could be used to infer the cell differentiation hierarchy and predict the epigenomes of differentiating intermediate progenitor cells.

Nucleosomes are typically depleted in regulatory regions such as promoters and enhancers, resulting in accessible chromatin [14]. Chromatin accessibility in gene-regulatory regions dynamically changes during cellular differentiation; moreover, the cell type-specific chromatin accessibility pattern is important for establishing and maintaining cellular identity [14, 15]. Chromatin accessibility not only reflects the expression status of genes [16], but also provides additional information to the transcriptome [17, 18]. Indeed, chromatin accessibility could represent cell types better than gene expression patterns in mammalian hematopoiesis [19, 20]. Therefore, estimating changes in chromatin accessibility during cell differentiation would be useful, especially for difficult-to-obtain progenitor cells.

The purpose of this study was to examine the feasibility of phylogenetic analysis based on genome-wide chromatin accessibility according to (1) tree topology, whether genome-wide chromatin accessibility data of differentiated cells can be used to infer the cell differentiation hierarchy and (2) ancestral state estimation, to predict the chromatin accessibility of differentiating intermediate progenitor cells. Mammalian hematopoietic differentiation is one of the best-studied systems due to its biological and medical importance, and the hierarchical structure along the course of differentiation is well known [21]. Additionally, many experimental efforts have been undertaken for obtaining epigenomes of not only terminally differentiated cells but also stem cells and progenitor cells (e.g. [22–25]). These known hierarchical structure and epigenomes of progenitor cells can be used as a reference (correct answer) to verify the computational inference, thus hematopoiesis provides a unique opportunity for inference. If phylogenetic analysis of epigenomes can be demonstrated for hematopoiesis, it offers the potential to explore other cell differentiation systems, such as solid tissues, which are more difficult to study.

## Main text

### Materials and methods

The chromatin accessibility information of candidate *cis*-regulatory elements (cCREs) of 18 murine hematopoietic cell types from the VISION project (Mouse_VISION_cCREs_2020_EpiGstates.txt) [20, 26] was obtained through https://usevision.org/data/ccre/cCREs/2020/. The project defines a cCRE as “a DNA segment assigned as a reproducible peak by ATAC-seq or DNase-seq that was not in a quiescent epigenetic state in all cell types” [20]. The cCREs have indexing information on the absence (0) or presence (1) of ATAC-seq/DNase-seq peaks. The cCREs vary in size with a median 265 bp and a mean 352 bp. Detailed methodology for identifying cCRE was described by Xiang et al. [20, 26]. In this study, a cCRE was treated as an independent “site” with binary (0/1) information. Among the 18 cell types, after excluding cell lines, stem cells (LSK, Lin^−^Sca1^+^Kit^+^ cells), three progenitor cells (CMP, common myeloid progenitor cells; GMP, granulocyte monocyte progenitor cells; and MEP, megakaryocyte erythrocyte progenitor cells) and eight differentiated cells (Ery, erythroblasts; iMK, immature megakaryocytes; Mon, monocytes; Neu, neutrophils; B, B cells; TCD4, CD4^+^ T cells; TCD8, CD8^+^ T cells; and NK, natural killer cells) were used.

Based on the binary information, a phylogenetic analysis was performed using neighbor-joining (NJ) [27], maximum parsimony (MP) [28], and maximum likelihood (ML) [29] methods. For the NJ method, the number of pairwise character (0/1) differences was used for calculating the distance matrix. For the MP method, characters (0/1) were treated as undirected characters (the cost of open is equal to that of close) and an exhaustive search was performed using PAUP version 4.0b10 [30]. For the ML method, six different models (BIN, BIN + I, BIN + I + G, and BIN + I + Rn where n = 4, 8, and 12, respectively) were computed where BIN represents binary data, I represents the ML estimates of the proportion of invariant sites, G represents the Gamma model of among-site rate heterogeneity with four categories, and Rn represents the free rate of among-site rate heterogeneity with n categories. Using the model with the lowest Akaike's Information Criterion, the best ML tree was searched using RAxML-NG version 1.1.0 [31]. For all three methods, branch support was evaluated based on 1000 bootstrap replicates.

Treelikeness was assessed using δ plots [32] with the delta.plot function of the ape package in R 4.3.0. For calculating δ_q_, LSK, B, TCD4, TCD8, and NK cells were used for the lymphoid lineage, whereas LSK, Neu, Mon, Ery, and iMK cells were used for the myeloid lineage.

An ancestral state of each site at each internal node was estimated based on MP and ML methods under the constraint of a fixed tree topology (see text). For the MP method, the ACCTRAN and DELTRAN algorithms were used to estimate the most parsimonious reconstruction [33]. For the ML method, marginal probabilities were used based on the best model described above. Ambiguous sites, estimated as “-“ by RAxML-NG, and stable sites, classified as STABLE (see text) in all lineages, were removed when calculating sensitivity and specificity. For both MP and ML methods, LSK was used as an outgroup.

To analyze characteristics of each cCRE, 27 epigenetic states defined by Xiang et al. [20] based on epigenetic marks of six histone modifications, CTCF binding, and nuclease accessibility were downloaded from https://usevision.org/data/mm10/IDEASmouseHem2019/segmentation/. The genomic positions were compared using the GenomicRanges package in R 4.3.0. To identify DNA motifs enriched in a specific cell lineage, findMotifsGenome.pl of HOMER [34] was used with default parameters.

### Results and discussion

**Fig. 1:**
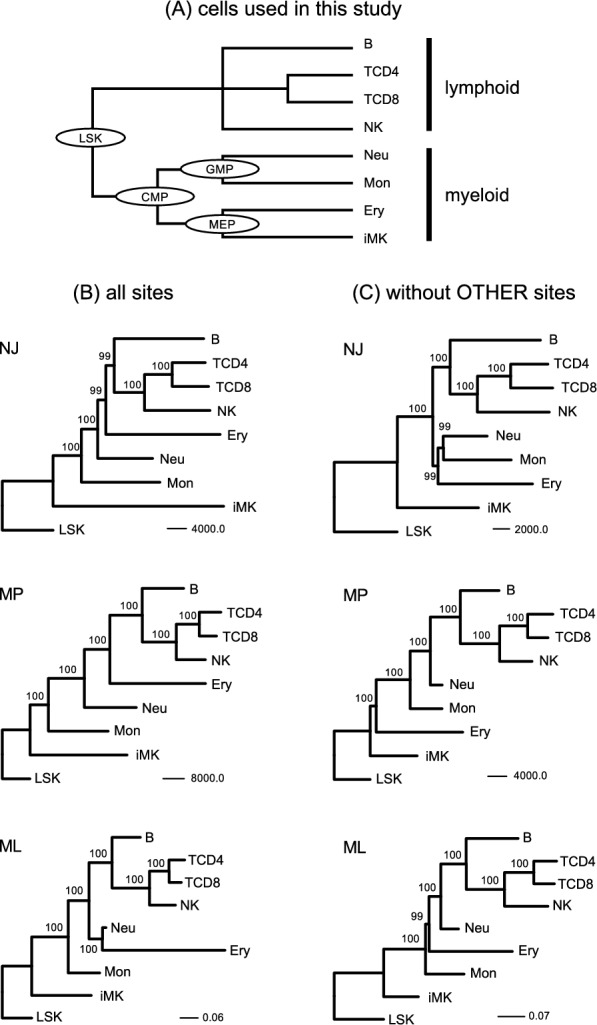
Inferred phylogenetic trees of hematopoietic cells. **A** Cells used in this study. Circles represent differentiating progenitor cells. Tree topology is based on the known hierarchical hematopoietic differentiation [20]. Inferred phylogenetic trees for all sites which are 205,019 sites (**B**) and all sites without OTHER sites which are 102,521 sites (**C**). Numbers on internal branches indicate bootstrap values. LSK was used as an outgroup

**Fig. 2:**
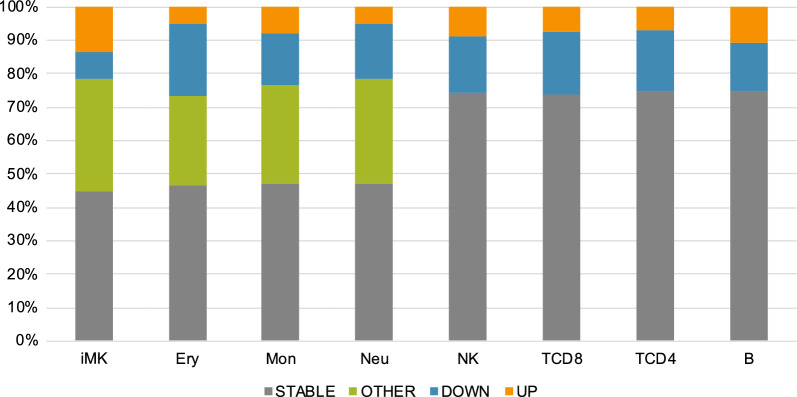
Classification of sites based on the changing pattern of open/closed chromatin states for each lineage. Each site was classified as STABLE, OTHER, DOWN, and UP according to the time-course changes of open chromatin signals for each lineage. STABLE includes both consistently open and closed sites. UP includes from closed to open changes, while DOWN includes from open to closed changes. Other sites are classified as OTHER

Chromatin accessibility data of murine hematopoietic cells were obtained from the VISION project, which integrates precise and comprehensive epigenetic states and provides valuable resources for murine hematopoiesis [20, 26]. The obtained accessible chromatin regions are 150–3659 bp in length and 77,695,128 bp in total. In this study, each region was treated as a site; a total of 205,019 sites were used. During hematopoiesis, hematopoietic stem cells produce lymphoid and myeloid lineages, consisting of a variety of differentiated cell types (Fig. 1A). Using this data, putative time-course changing patterns of open/closed chromatin were examined in each of eight lineages (from LSK to B, TCD4, TCD8, NK, Neu, Mon, Ery, and iMK) during hematopoiesis. Sites were classified into four categories as follows: “STABLE,” “UP,” “DOWN,” and “OTHER” depending on their changes along the differentiation path: STABLE sites are consistently open or closed; UP or DOWN sites are gradually open or closed during hematopoiesis, respectively; the rest of sites were classified as OTHER. As a result, UP sites and DOWN sites accounted for 4.9–14% and 7.8–22%, respectively (Fig. 2). These sites could be suitable for phylogenetic analysis (see below). On the other hand, OTHER sites comprised 27%–34% in myeloid lineages (Fig. 2). This proportion of OTHER sites were larger than those previously reported for DNA methylation [10]. When considering all lineages, about half of the sites contained OTHER sites in ≥ 1 lineage (Additional file 1: Figure S1). The difference in the proportion of OTHER sites between chromatin accessibility and DNA methylation may reflect the feature of each epigenome, where chromatin accessibility showed a strong positive correlation with gene expression, whereas DNA methylation was relatively stable [17], whose mechanism of enzymatic maintenance is well known [35]. Note that no sites could be classified as OTHER for the lymphoid lineages because chromatin accessibility data were not available for progenitor cells, and the time course of this lineage only included one step (Fig. 1A).

When using chromatin accessibility data to reconstruct the cell differentiation hierarchy in phylogenetic analysis, UP and DOWN sites could contain useful information. Conversely, STABLE sites contain no information while OTHER sites may contain too many multiple changes in a site and/or homoplasious changes in multiple lineages, which sometimes hinders correct phylogenetic inferences for the MP method [36] and possibly for the ML method because incorporating the appropriate model can be difficult for these cases. Based on the chromatin accessibility data, phylogenetic analysis was performed with NJ, MP, and ML methods. Open (1) or closed (0) chromatin states were treated as binary information, and each chromatin region was treated as a site. For the ML method, the best-fit model, BIN + F0 + I + R4, was used.

When all sites were included in the analysis, the lymphocyte lineage was separated with high bootstrap values with all three methods (Fig. 1B). On the other hand, the diversification pattern of the myeloid lineage was different from the known topology (Fig. 1A). Removing the OTHER sites improved the monophyly of neutrophils and monocytes with the NJ method but not with the other methods (Fig. 1C). Furthermore, removing iMK could recover the monophyly of the myeloid lineage in the ML method and could reconstruct the known topology in the NJ method (Additional file 2: Figure S2). The iMK contained more UP sites than other cells (Fig. 2), which might cause the long branch of iMK. These results may reflect a limitation of phylogenetic analysis, which can be affected by homoplasious sites, long branches [36], and dependencies between sites [37]. Another possibility is heterogeneity in cell populations, recently revealed by new technologies [21], which implicated multiple differentiation paths. In fact, megakaryocytes could be differentiated directly from stem cells [38], a finding consistent with the inferred tree (Fig. 1B and C). In addition, since red blood cells and platelets lack DNA, their progenitors (erythrocytes and immature megakaryocytes) were used in this study, which may cause some problems. When treelikeness was compared between the lymphoid and myeloid lineages, lymphoid lineage exhibited lower δ_q_ (Additional file 3: Figure S3), which ranges from 0 (perfectly treelike) to 1 [32]. It appears that chromatin accessibility of myeloid lineage contains less information suitable for phylogenetic analysis. Removing OTHER sites increased the treelikeness, consistent with results of the phylogenetic analysis (Fig. 1B and C). Therefore, selecting the appropriate sites is important for applying phylogenetic analysis based on genome-wide chromatin accessibility for inferring cell differentiation processes.

**Fig. 3:**
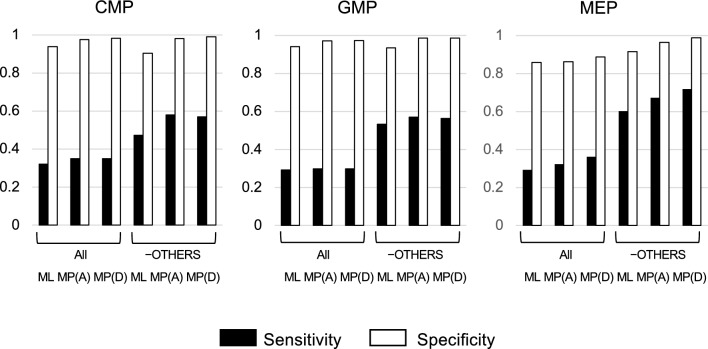
Prediction of open chromatin regions in differentiating progenitor cells. Black bars indicate sensitivity and white bars specificity. **A** and **D** represent ACCTRAN and DELTRAN, respectively

Phylogenetic analysis also allows for estimating the ancestral states of internal nodes. Therefore, we next predicted the open/closed chromatin states of internal nodes, which correspond to differentiating progenitor cells (CMP, GMP, and MEP), and compared the predicted states of internal nodes with those of progenitor cells obtained from the VISION project [20, 26]. For this analysis, the STABLE sites on all lineages were removed; thus, a total of 175,083 sites were used. The ancestral states of the internal nodes were estimated using MP (ACCTRAN and DELTRAN) and ML (best-fit BIN + F0 + I + R4 model) methods under the topological constraint of the known tree (Fig. 1A) and the consistently inferred lymphoid topology of (B, ((TCD4, TCD8), NK)) (Fig. 1B and C). Then, comparison of the chromatin states of the predicted internal nodes with those of progenitor cells was used to calculate the sensitivity and specificity (Fig. 3). For the ML method, the calculations were performed by removing ambiguously estimated sites (4188, 4026, 4188 sites for the internal nodes corresponding to CMP, GMP, and MEP, respectively).

As a result, both MP and ML showed good specificities, between 0.86 and 0.98, depending on cell types. On the other hand, both methods showed low sensitivities for all cell types, ranging between 0.29 and 0.36, possibly due to false negatives from OTHER sites (Fig. 2). In fact, when the OTHER sites (102,498 sites) were removed, the specificity and sensitivity was improved to 0.90–0.99 and 0.47–0.72, respectively. Note that even among UP and DOWN sites, some sites cannot be correctly predicted in principle. For example, stem and progenitor cell specific/unique open regions are impossible to infer by phylogenetic analysis. When DNA methylation data were used (materials and methods were described in [10]), specificities and sensitivities ranged between 0.61–0.96 and 0.72–0.92, respectively, indicating better predictability, especially for sensitivity. This difference between DNA methylation and chromatin accessibility may reflect the dynamic feature of chromatin accessibility compared with DNA methylation [17], as discussed in Fig. 2.

Finally, the biological implications from the inferences based on this phylogenetic analysis were explored in two aspects. First, epigenetic marks for each site class (STABLE/DOWN/UP/OTHER) were examined (Additional file 4: Figure S4). Xiang et al. assigned “epigenetic states, which are common combination of epigenetic features” [20] based on six histone modifications, CTCF binding, and nuclease accessibility of mouse hematopoietic cells. Based on this information, regions overlapping with these epigenetic states were analyzed for each site class of each myeloid cell (Neu, Mon, Ery, and iMK). As a result, DOWN sites exhibited a decrease, while UP sites exhibited an increase in epigenetic state 9, indicating high levels of nuclease accessibility, as expected, except for UP sites of erythrocytes. Interestingly, OTHER sites showed a progenitor-specific elevation of this state, suggesting progenitor-specific gene regulation. In addition, increase in epigenetic states of active promoter and enhancer signature, such as 12 and 21 [20], was observed in the UP sites. Second, enrichment of the transcription factor binding motifs was searched for genomic regions that showed lineage-specific changes of open/closed chromatin states, as demonstrated by Xiang et al. [20]. There were 3072, 44, and 184 sites with a change from open to closed in the lineages from LSK to CMP, from CMP to MEP, and from CMP to GMP, respectively, whereas 521, 129, and 53 sites with a change from closed to open in the lineages from LSK to CMP, from CMP to MEP, and from CMP to GMP, respectively, where no changes were observed in other cell lineages of myeloid. When DNA motifs were searched in the most prominent 3072 sites with open-to-closed changes in the lineage from LSK to CMP, it was found that 12 DNA motifs were statistically enriched in these regions (Additional file 5: Figure S5). Of these, four motifs, including Runx1 and IRF1 binding motifs, are involved in lymphoid cell lineage determination [24], which is consistent with the closed states at the branching point of myeloid cell lineage. PBX2, NF1, NF-E2, CREB, and Tlx-1 are related to hematopoietic cells (eg. [39–43]). Other motifs might contain candidates for further studies.

In summary, the present phylogenetic analysis of chromatin accessibility data could partially infer the cell differentiation hierarchy of murine hematopoiesis. The epigenomes of progenitor cells could be estimated with high specificity but with low sensitivity, possibly due to the characteristics of chromatin accessibility, which is closely related to gene expression [17] and reflects diverse cell types [19, 20]. Changes in chromatin accessibility during cell differentiation include important changes involved in the divergence of cell lineages. Therefore, the results presented in this study suggest that the phylogenetic analysis of chromatin accessibility may provide additional information on cell differentiation.

### Limitations

This study is based on murine hematopoiesis; thus, it is unclear whether the present findings are applicable to other species and/or cell types. Based on transcriptomic data, hierarchical differentiation was observed for many cell types other than hematopoietic cells [44]; thus, it is interesting to see whether it can be applied to other cell types. In addition, a traditional hierarchical differentiation of hematopoiesis was assumed in the present study (Fig. 1A). However, this model has recently been challenged by new evidence of a continuous model of hematopoiesis [21]. These need to be further studied in the future.

### Supplementary Information

**Additional file 1: Figure S1.** Classification of sites for all lineages. Each site was classified as STABLE, DOWN, UP, and OTHER depending on the time-course changes in chromatin states through all lineages and then summarized.

**Additional file 2: Figure S2.** Inferred phylogenetic trees of hematopoietic cells without iMK. Inferred phylogenetic trees for all sites, which include 205,019 sites (A) and all sites without OTHER sites, which include 102,521 sites (B). Numbers on the internal branches indicate bootstrap values. LSK was used as an outgroup.

**Additional file 3: Figure S3.** plots for lymphoid and myeloid lineages. Treelikeness was assessed based on all (205,019) sites (A) and all sites without OTHER (102,521) sites (B).

**Additional file 4: Figure S4.** Epigenetic states for each site class. (A) For each site class of iMK, Ery, Mon, and Neu, total length of the regions with each epigenetic state for each cell was plotted. To highlight the differences, epigenetic state 0 (all the epigenomes are quiescence), which was most abundant for all cells, was not displayed. (B) Heatmap of each epigenetic state, which is based on the information provided by the VISION project (https://usevision.org/data/mm10/IDEASmouseHem2019/ideasVisionV20p8Seg.statesig.para).

**Additional file 5: Figure S5.** DNA motifs enriched in the region with the change from open to closed chromatin in the lineage from LSK to CMP. (A) Hematopoietic differentiation and transcription factors regulating each cell lineage (modified from Lara-Astiaso et al. 2014 [24]). Transcription factors whose motifs were found enriched are highlighted. (B) The results of DNA motif discovery. TFs appeared in (A) are marked with asterisks.

## Data Availability

All data generated or analyzed during this study were included in this published article and its supplementary information files.

## Abbreviations

LSK: Lin^−^Sca1^+^Kit^+^, which includes hematopoietic stem cells and multipotent progenitor cells
CMP: Common myeloid progenitor cells
GMP: Granulocyte monocyte progenitor cells
MEP: Megakaryocyte erythrocyte progenitor cells
Ery: Erythroblasts
iMK: Immature megakaryocytes
Mon: Monocytes
Neu: Neutrophils
B: B cells
TCD4: CD4^+^ T cells
TCD8: CD8^+^ T cells
NK: Natural killer cells
