# Supplementary material for: Inferring chromatin accessibility during murine hematopoiesis through phylogenetic analysis
Source: BMC Res Notes. 2023 Sep 19;16:222. doi: 10.1186/s13104-023-06507-8 (PMC10507877; doi:10.1186/s13104-023-06507-8)
Supplement: Supplementary file 1 — Additional file 1: Figure S1. Classification of sites for all lineages. Each site was classified as STABLE, DOWN, UP, and OTHER depending on the time-course changes in chromatin states through all lineages and then summarized. [file 13104_2023_6507_MOESM1_ESM.pptx]

## Slide 1
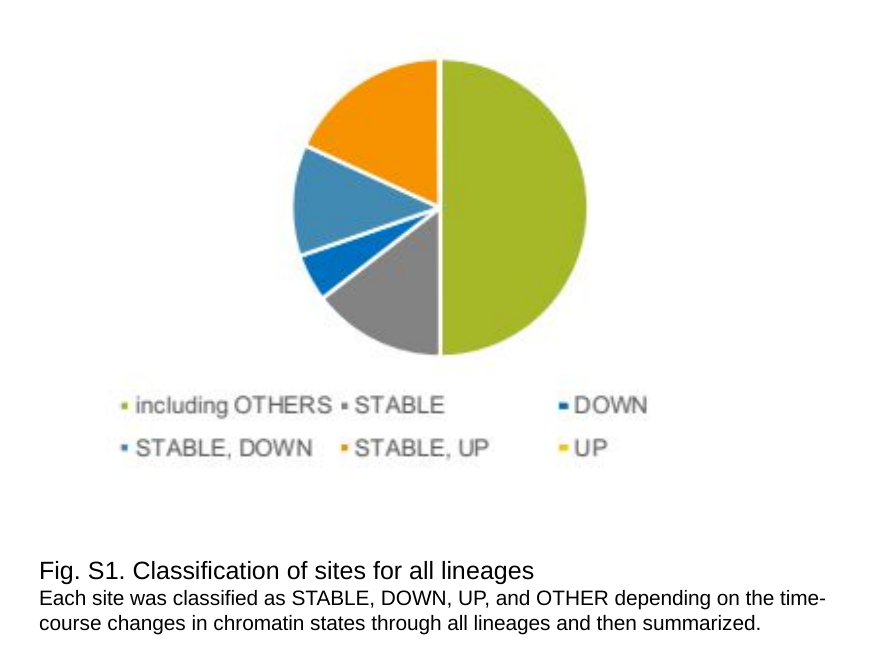

Fig. S1. Classification of sites for all lineages
Each site was classified as STABLE, DOWN, UP, and OTHER depending on the time-course changes in chromatin states through all lineages and then summarized.
