# Supplementary material for: Inferring chromatin accessibility during murine hematopoiesis through phylogenetic analysis
Source: BMC Res Notes. 2023 Sep 19;16:222. doi: 10.1186/s13104-023-06507-8 (PMC10507877; doi:10.1186/s13104-023-06507-8)
Supplement: Supplementary file 2 — Additional file 2: Figure S2. Inferred phylogenetic trees of hematopoietic cells without iMK. Inferred phylogenetic trees for all sites, which include 205,019 sites (A) and all sites without OTHER sites, which include 102,521 sites (B). Numbers on the internal branches indicate bootstrap values. LSK was used as an outgroup. [file 13104_2023_6507_MOESM2_ESM.pptx]

## Slide 1
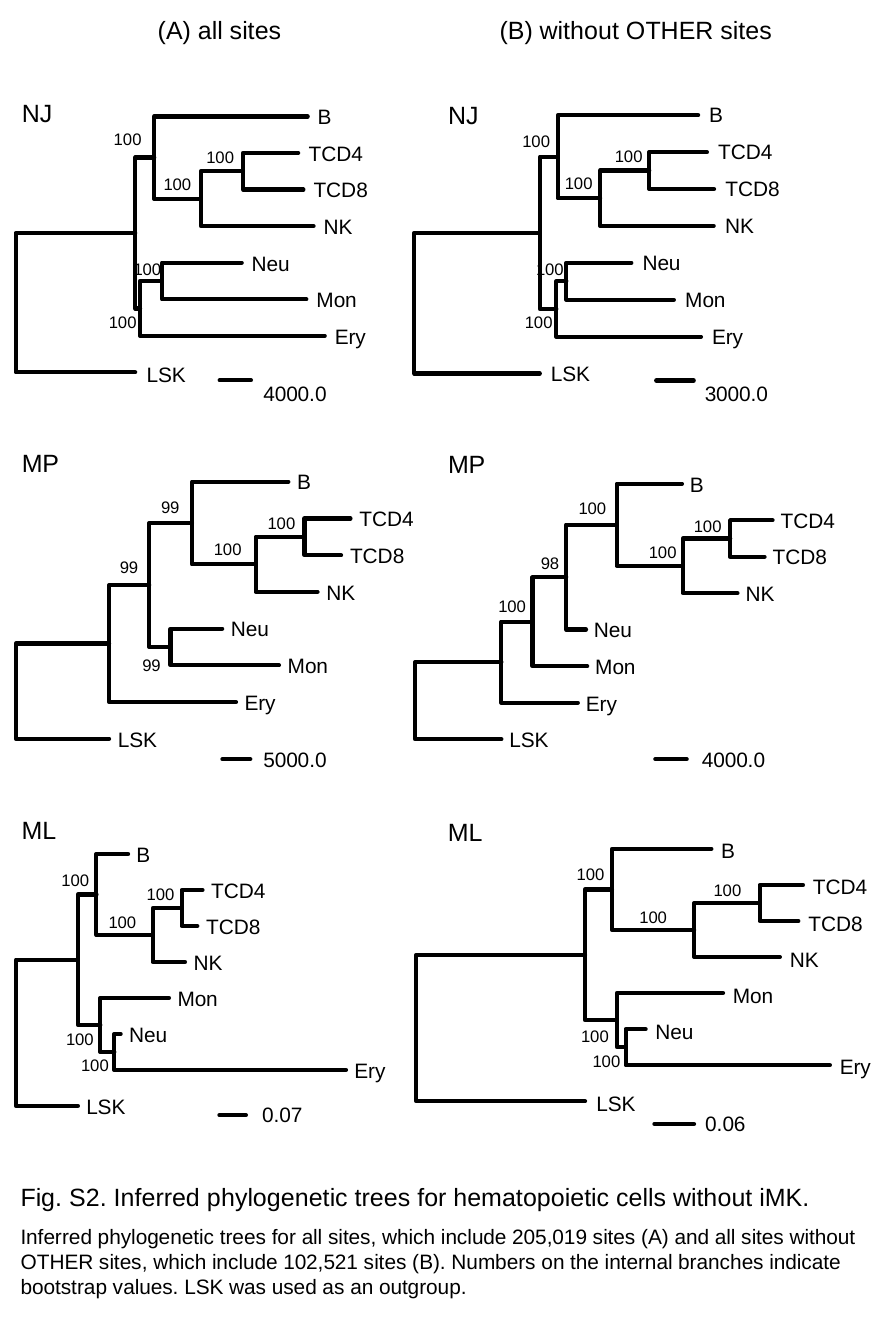

(A) all sites
(B) without OTHER sites
NJ
NJ
B
B
100
100
TCD4
TCD4
100
100
100
100
TCD8
TCD8
NK
NK
Neu
Neu
100
100
Mon
Mon
100
100
Ery
Ery
LSK
LSK
4000.0
3000.0
MP
MP
B
B
99
100
TCD4
TCD4
100
100
100
100
TCD8
TCD8
98
99
NK
NK
100
Neu
Neu
Mon
Mon
99
Ery
Ery
LSK
LSK
5000.0
4000.0
ML
ML
B
B
100
100
TCD4
TCD4
100
100
100
TCD8
100
TCD8
NK
NK
Mon
Mon
Neu
Neu
100
100
100
Ery
100
Ery
LSK
LSK
0.07
0.06
Fig. S2. Inferred phylogenetic trees for hematopoietic cells without iMK.
Inferred phylogenetic trees for all sites, which include 205,019 sites (A) and all sites without OTHER sites, which include 102,521 sites (B). Numbers on the internal branches indicate bootstrap values. LSK was used as an outgroup.
