# Supplementary material for: Inferring chromatin accessibility during murine hematopoiesis through phylogenetic analysis
Source: BMC Res Notes. 2023 Sep 19;16:222. doi: 10.1186/s13104-023-06507-8 (PMC10507877; doi:10.1186/s13104-023-06507-8)
Supplement: Supplementary file 3 — Additional file 3: Figure S3. plots for lymphoid and myeloid lineages. Treelikeness was assessed based on all (205,019) sites (A) and all sites without OTHER (102,521) sites (B). [file 13104_2023_6507_MOESM3_ESM.pptx]

## Slide 1
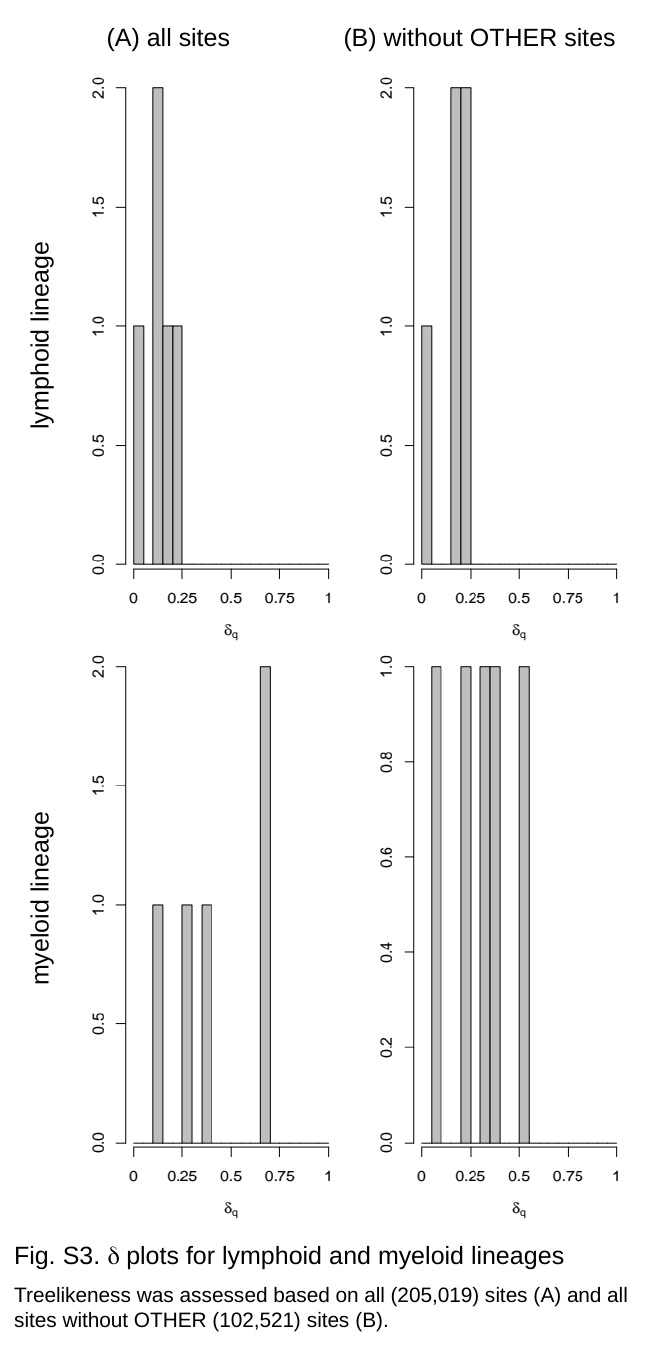

(A) all sites
(B) without OTHER sites
lymphoid lineage
myeloid lineage
Fig. S3. d plots for lymphoid and myeloid lineages
Treelikeness was assessed based on all (205,019) sites (A) and all sites without OTHER (102,521) sites (B).
