# Supplementary material for: Inferring chromatin accessibility during murine hematopoiesis through phylogenetic analysis
Source: BMC Res Notes. 2023 Sep 19;16:222. doi: 10.1186/s13104-023-06507-8 (PMC10507877; doi:10.1186/s13104-023-06507-8)
Supplement: Supplementary file 4 — Additional file 4: Figure S4. Epigenetic states for each site class. (A) For each site class of iMK, Ery, Mon, and Neu, total length of the regions with each epigenetic state for each cell was plotted. To highlight the differences, epigenetic state 0 (all the epigenomes are quiescence), which was most abundant for all cells, was not displayed. (B) Heatmap of each epigenetic state, which is based on the information provided by the VISION project (https://usevision.org/data/mm10/IDEASmouseHem2019/ideasVisionV20p8Seg.statesig.para). [file 13104_2023_6507_MOESM4_ESM.pptx]

## Slide 1
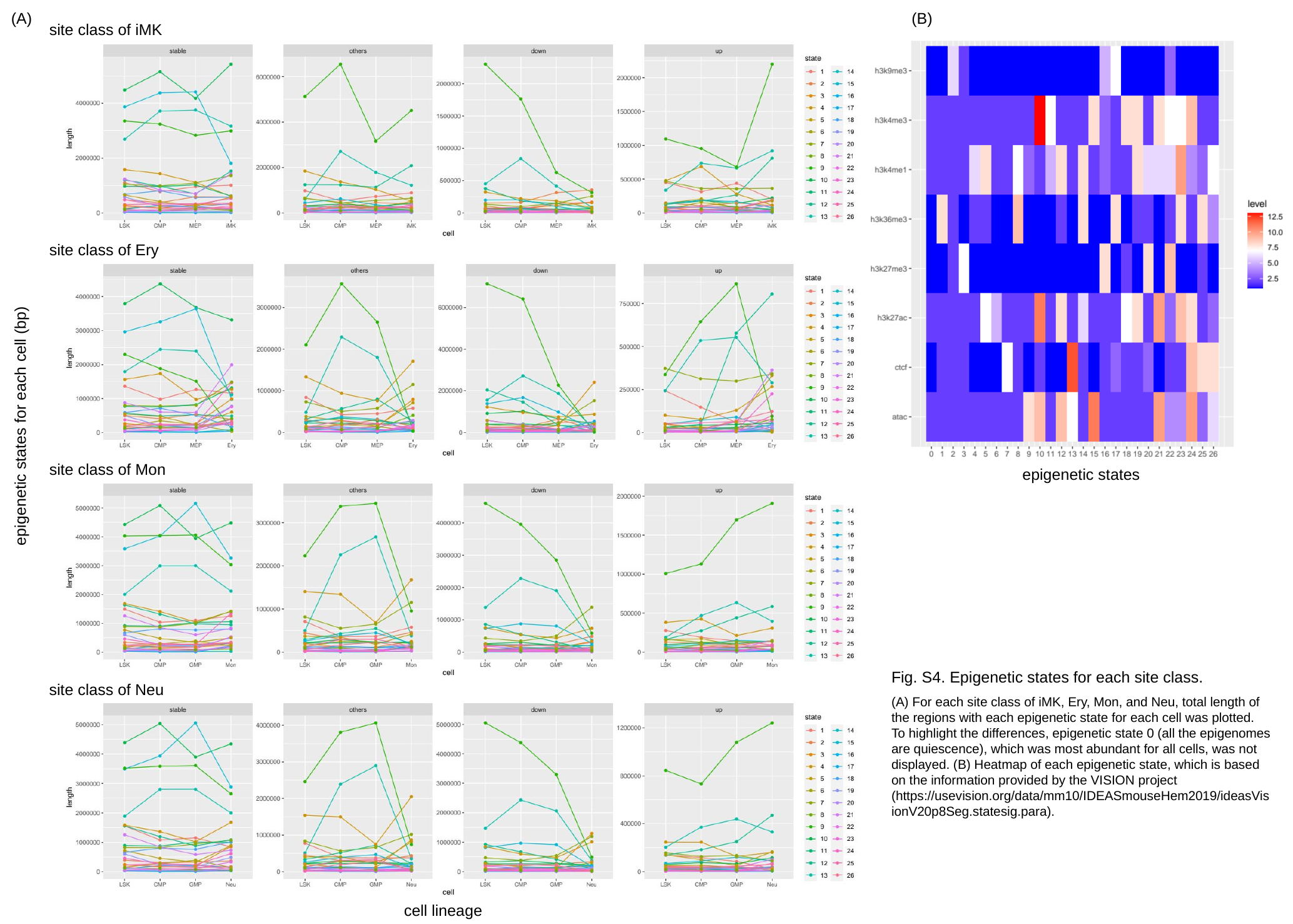

(A)
(B)
site class of iMK
site class of Ery
 epigenetic states for each cell (bp)
site class of Mon
epigenetic states
Fig. S4. Epigenetic states for each site class.
(A) For each site class of iMK, Ery, Mon, and Neu, total length of the regions with each epigenetic state for each cell was plotted. To highlight the differences, epigenetic state 0 (all the epigenomes are quiescence), which was most abundant for all cells, was not displayed. (B) Heatmap of each epigenetic state, which is based on the information provided by the VISION project (https://usevision.org/data/mm10/IDEASmouseHem2019/ideasVisionV20p8Seg.statesig.para).
site class of Neu
cell lineage
