# Supplementary material for: Inferring chromatin accessibility during murine hematopoiesis through phylogenetic analysis
Source: BMC Res Notes. 2023 Sep 19;16:222. doi: 10.1186/s13104-023-06507-8 (PMC10507877; doi:10.1186/s13104-023-06507-8)
Supplement: Supplementary file 5 — Additional file 5: Figure S5. DNA motifs enriched in the region with the change from open to closed chromatin in the lineage from LSK to CMP. (A) Hematopoietic differentiation and transcription factors regulating each cell lineage (modified from Lara-Astiaso et al. 2014 [24]). Transcription factors whose motifs were found enriched are highlighted. (B) The results of DNA motif discovery. TFs appeared in (A) are marked with asterisks. [file 13104_2023_6507_MOESM5_ESM.pptx]

## Slide 1
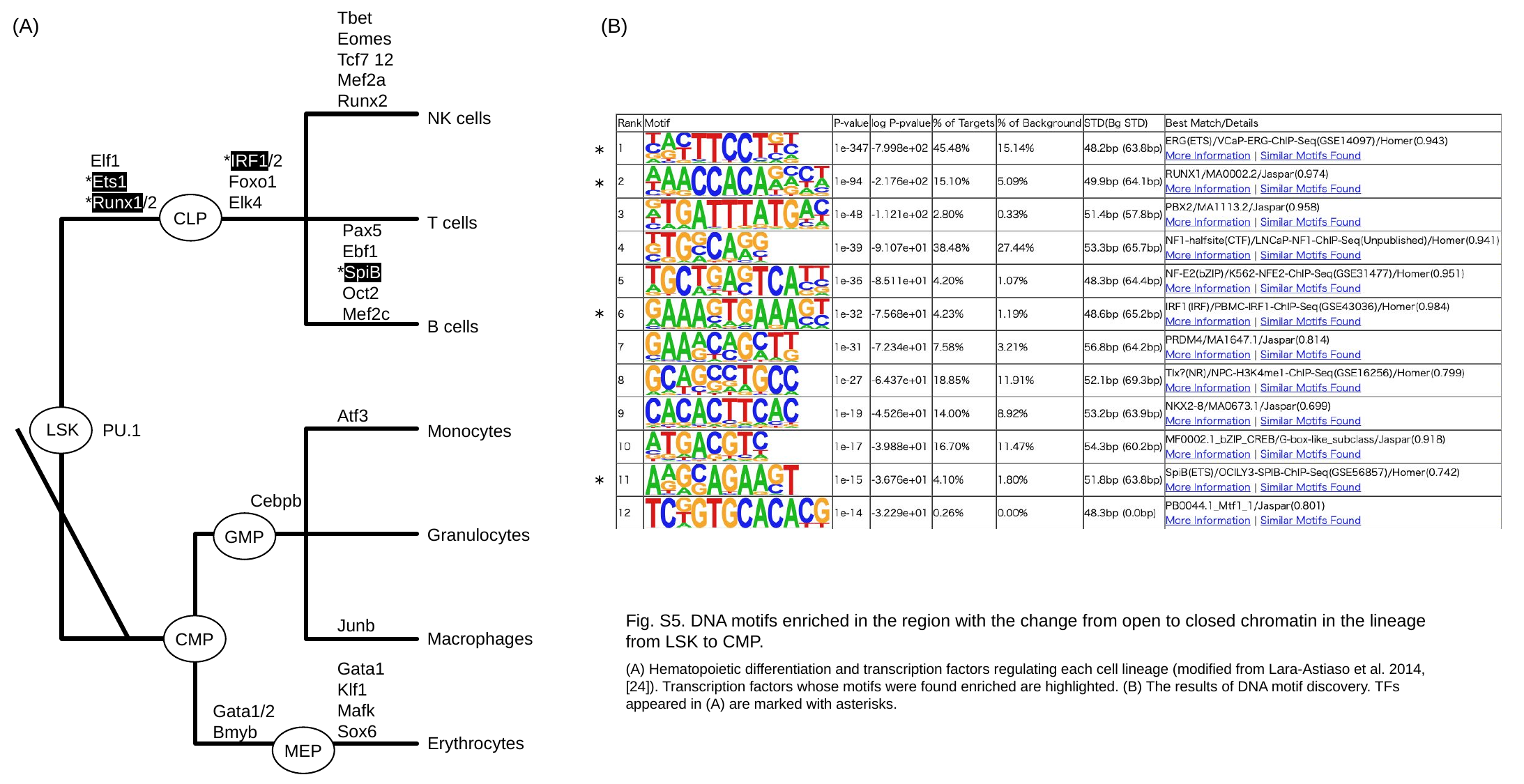

Tbet
Eomes
Tcf7 12
Mef2a
Runx2
NK cells
 Elf1
*Ets1
*Runx1/2
*IRF1/2
 Foxo1
 Elk4
CLP
T cells
 Pax5
 Ebf1
*SpiB
 Oct2
 Mef2c
B cells
Atf3
PU.1
Monocytes
Cebpb
Granulocytes
GMP
Junb
Macrophages
CMP
Gata1
Klf1
Mafk
Sox6
Gata1/2
Bmyb
Erythrocytes
MEP
(A)
(B)
*
*
*
*
LSK
Fig. S5. DNA motifs enriched in the region with the change from open to closed chromatin in the lineage from LSK to CMP.
(A) Hematopoietic differentiation and transcription factors regulating each cell lineage (modified from Lara-Astiaso et al. 2014, [24]). Transcription factors whose motifs were found enriched are highlighted. (B) The results of DNA motif discovery. TFs appeared in (A) are marked with asterisks.
